# Iron Oxide Nanoparticles: Selectively Targeting Melanoma Cells In Vitro by Inducing DNA Damage via H2AX Phosphorylation and Hindering Proliferation through ERK Dephosphorylation

**DOI:** 10.3390/pharmaceutics16040527

**Published:** 2024-04-11

**Authors:** Danai E. Prokopiou, Anastasia Chillà, Francesca Margheri, Gabriella Fibbi, Anna Laurenzana, Eleni K. Efthimiadou

**Affiliations:** 1 Inorganic Chemistry Laboratory, Chemistry Department, National and Kapodistrian University of Athens, Panepistimiopolis, 157 71 Zografou, Greece; dprokop@chem.uoa.gr; 2 Institute of Nanoscience and Nanotechnology, NCSR “Demokritos”, 153 41 Athens, Greece; 3 Department of Experimental and Clinical Biomedical Sciences “Mario Serio”, University of Florence, Viale G. B. Morgagni, 50, 50134 Florence, Italy; anastasia.chilla@unifi.it (A.C.); francesca.margheri@unifi.it (F.M.); gabriella.fibbi@unifi.it (G.F.)

**Keywords:** iron oxide magnetic nanoparticles, melanoma, in vitro magnetic hyperthermia, western blot

## Abstract

This study investigates the distinctive characteristics of iron oxide magnetic nanoparticles (mNPs) and their potential application in cancer therapy, focusing on melanoma. Three types of mNPs, pre-validated for safety, underwent molecular analysis to uncover the activated signaling pathways in melanoma cells. Using the Western blot technique, the study revealed that mNPs induce cytotoxicity, hinder proliferation through ERK1/2 dephosphorylation, and prompt proapoptotic effects, including DNA damage by inducing H2AX phosphorylation. Additionally, in vitro magnetic hyperthermia notably enhanced cellular damage in melanoma cells. Moreover, the quantification of intracellular iron levels through Inductively Coupled Plasma Mass Spectrometry (ICP-MS) analysis unveils the precise dosage required to induce cellular damage effectively. These compelling findings not only shed light on the therapeutic potential of mNPs in melanoma treatment but also open exciting avenues for future research, heralding a new era in the development of targeted and effective cancer therapies. Indeed, by discerning the effective dose, our approach becomes instrumental in optimizing the therapeutic utilization of iron oxide magnetic nanoparticles, enabling the induction of precisely targeted and controlled cellular responses.

## 1. Introduction

Iron oxide magnetic nanoparticles (mNPs) are at the epicenter of scientific research due to their broad spectrum of applications in nanomedicine [1].

In this context, mNPs have paved their way in cancer treatment as theranostic materials, as well as into magnetic resonance imaging as colloidal mediators for magnetic hyperthermia and drug delivery [2,3], due to their interesting physical and chemical properties, their colloidal stability, and facile synthesis [4]. Magnetic hyperthermia (MH) is indeed an important nanostructure-based approach in cancer therapy. When exposed to an alternating magnetic field (AMF), the mNPs rapidly align with the AMF, leading to a phenomenon known as hysteresis. As a result, the magnetic energy absorbed by the nanoparticles is converted into heat [5]. The generated heat causes an increase in temperature within the tumor, selectively affecting the cancer cells while minimizing damage to the surrounding healthy tissues.

The localized heating induced by MH can have several effects on tumors. Firstly, the elevated temperature can directly induce cell death by triggering apoptosis or necrosis of cancer cells. Secondly, the heat can disrupt the tumor microenvironment, damaging blood vessels and impairing nutrient and oxygen supply to the tumor, thereby further inhibiting tumor growth. Additionally, the increased temperature can stimulate an immune response, leading to the activation of immune cells and enhancing the body’s natural defense mechanisms against cancer [6,7].

Despite an ever-increasing volume of data confirming mNPs therapeutic efficiency, studies are still in the early stage of clinical evaluation. Among these data, NP biocompatibility, toxicity, and activation of an immune response are challenges that need to be addressed. Among different parameters NP size, shape, and surface charge (NPs) can play a central role in cellular internalization and distribution and, thus, in their actual efficacy and toxicity [8,9]. Moreover, surface modification of magnetic nanoparticles (mNPs) with specific biological molecules is a promising strategy to enhance the selectivity and efficiency of drug delivery systems while minimizing the exposure of cytotoxic agents to non-target tissues. However, achieving successful and safe in vivo applications of mNPs requires a profound understanding of their interactions with cells at both ultrastructural and molecular levels [10,11,12].

In this regard, healthy cells are used to evaluate the toxicity as well as therapeutic efficacy of mNPs, providing significant information for the understanding of cell–nanoparticle interactions, a key step before in vivo experimentation. In our previous work, we have synthesized bare mNPs and we have modified their surface with tri-sodium citrate and sodium borohydride as reducing agents to improve their properties such as colloidal behavior. The in vitro results confirmed mNP biocompatibility and safety by enabling further investigation of their theranostic potential [13]. In the current study, we explore the signaling pathways selectively triggered and modulated by mNPs in the “bare” or non-functionalized state in cancer and healthy cells. Remarkably, we observe that all three kinds of mNPs tested do not affect cell viability of normal human keratinocyte NCTC [14], but rather exploit selective cytotoxic, antiproliferative, and proapoptotic effects, indicating the potential anticancer activity of mNPs per se. As expected, mNP-mediated hyperthermia treatment on melanoma cells was found to enhance cellular damage.

## 2. Materials and Methods

### 2.1. Materials

The mNPs that are studied in this paper were previously synthesized using the co-precipitation method [13].

### 2.2. Cell Culture

A375-M6 melanoma cells (M6) were cultured in the Florence laboratory as described in the Laurenzana et al.’s protocol [15]. NCTC 2544 human keratinocytes were used as healthy cell control and purchased from the American Type Culture Collection (ATCC, Rockville, MD, USA) and cultured as previously described [13].

### 2.3. Evaluation of Cell Viability

The viability of the A375-M6 cells was evaluated by trypan blue staining protocol as previously described [15]. Briefly, 1.5 × 10^5^ cells were seeded in 6-well plates and allowed to grow overnight. After overnight growing, the cells were treated with mNPs at different concentrations for 24 h. A total of 20 μL of cell suspension was transferred to a 1.5 mL clear eppendorf and incubated for 3 min at room temperature (rt) with an equal volume of trypan blue solution (0.4% *w*/*v* in 0.81% NaCl and 0.06% *w*/*v* dibasic potassium phosphate). Stained cells were counted separately through a hemocytometer under a microscope. For statistical reasons, three independent experiments were conducted, with the mean plotted in Section 3.3 [16].

### 2.4. Confocal Microscopy Analysis

**Figure 1:**
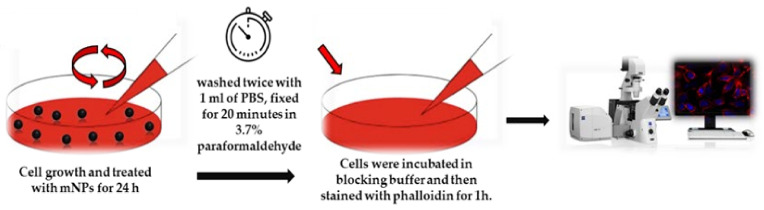
Procedure for confocal microscopy analysis.

For confocal imaging analysis, we followed the protocol published before [17]. Briefly, 1 × 10^5^ cells were grown on glass coverslips with polylysine, incubated with mNPs for 24 h, and then washed twice with 1 mL of PBS, fixed for 20 min in 3.7% paraformaldehyde in PBS, and permeabilized with 0.1% Triton X-100 in PBS for 5 min. Cells were treated in a blocking buffer (3% BSA and 0.1% Triton X-100 in PBS) for 1h at rtand stained with phalloidin for 1 h. Hoechst33342 dye (Blue) (10 µg/mL) (Invitrogen, Waltham, MA, USA) was used for nuclei staining for 15 min incubation at rt. An anti-fade mounting medium (Biomeda, Foster City, CA, USA) was used for observing the labeled cells under a Bio-Rad MRC 1024 ES Confocal Laser Scanning Microscope (Bio-Rad, Hercules, CA, USA) equipped with a 15 mW Krypton/Argon laser source for fluorescence measurements (Figure 1). Cells were examined with a Nikon Plan Apo X60oil immersion objective using an excitation wavelength appropriate for Alexa 488 (495 nm). A series of optical sections (XY: 512 × 512 pixels) were then taken through the depth of the cells with a thickness of 1 µm at intervals of 0.8 µm (Z step). A single composite image was obtained by the superimposition of twenty optical sections for each sample [17].

### 2.5. Western Blot

After mNP incubation, the cell pellet was treated for 30 min on ice with a lysis buffer containing RIPA (50 mM Tris (pH 7.4), 150 mM NaCl, 1% Triton X-100, 1% sodium deoxycholate, 0.1% SDS, 5 mM EDTA) and a proteinase inhibitor cocktail (Roche, Mannheim, Germany). Lysates were then centrifuged at 14,000 r.p.m. for 10 min. Aliquots of lysates (40 μg) of A375-M6 cells and NCTC 2544 were subjected to western blotting. The primary antibodies were anti-γH2AX (Cell Signaling Technology, Danvers, MA, USA), anti-pERK (Cell Signaling Technology, Danvers, MA, USA), and anti-GAPDH (Abcam, Cambridge, UK) used as a loading control. Membranes were incubated in a blocking solution consisting of phosphate-buffered saline (PBS)/Odyssey Blocking Buffer 1:1 (PBS/OBB) (Lycor Bioscience, Lincoln, NE, USA) for 1 h at rt. Membranes were then incubated overnight at 4 °C with the appropriate antibody, washed four times with PBS-Tween 0.1% solution, and probed with the secondary Anti-Rabbit IgG (whole molecule)–Peroxidase antibody (Sigma, St. Louis, MO, USA, Cat#A0545). The ECL procedure was employed for development.

### 2.6. TEM Analysis of mNPs-Enriched A375-M6

A total of 1.5 × 10^5^ A375-M6 cells were seeded in 6-well plates and allowed to reach 70% confluence. After 24 h, 2 mL of mNP suspension was added to each well and the cells were treated for 24 h. After that, the cells were incubated with trypsin for 10 min and their suspension was centrifuged at 1000 rpm for 5 min in 1.5 mL eppendorfs. The isolated cell pellet was fixed and dehydrated by glutaraldehyde and OsO_4_, respectively (isotonic 4% and 1%). The fixed cells were embedded in Epon epoxy resin (Fluka, Buchs, Switzerland) for TEM imaging. Ultrathin sections were stained with aqueous uranyl acetate and alkaline bismuth subnitrate, viewed, and photographed under a JEM 1010 transmission electron microscope (Jeol, Tokyo, Japan) equipped with a MegaView III high-resolution digital camera and imaging software (Jeol).

### 2.7. Cell Cycle Analysis

Cell cycle analysis was performed through the propidium iodide (PI) staining method [18]. According to this methodology, the sub-G1 cell population was analyzed. A total of 1.5 × 10^5^ cells were treated with mNPs, as previously mentioned, and the pellet was harvested for 24 h after centrifugation. The cells were washed with PBS two times and stained with PI for 30 min in the dark at rt (100 μg/mL PI, 20 μg/mL RNase A, 1 mg/mL trisodium citrate, and 0.3% (*v*/*v*) Triton X-100). The stained cells were analyzed by flow cytometry (BD-FACS Canto) using red propidium–DNA fluorescence.

### 2.8. ICP-AES Analysis

The iron concentration internalized in cells was calculated by a Varian 720-ES Inductively Coupled Plasma Atomic Emission Spectrometer (ICP-AES) equipped with a CETAC U5000 AT+ ultrasonic nebulizer, the latter allowing for increased method sensitivity. Cellular pellets were digested in a thermo-reactor at 80 °C for 6 h with 500 µL of concentrated supra pure HNO_3_ obtained by sub-boiling distillation. All samples were diluted to a final volume of 5.0 mL and spiked with 1 ppm of Ge which is used as an internal standard before the analysis. Preparation of internal standards was achieved by gravimetric serial dilution from a commercial standard solution of Fe at 1000 mg/L. The wavelength used for Fe determination was 238.204 nm, whereas for Ge the line at 209.426 nm was used. The operating conditions were optimized to obtain maximum signal intensity, and between each sample, a rinse solution of 1% *v*/*v* HNO_3_ suprapur grade was used to avoid any “memory effect” [19].

### 2.9. In Vitro Magnetic Hyperthermia Treatment (MHT) on A375-M6 Melanoma Cancer Cells

A total of 1.5 × 10^5^ A375-M6 cancer cells were seeded in a 6-well plate and left to be confluent for 24 h. Then, the cells were treated with trypsin to promote detachment and the suspension was centrifuged at 3000 rpm for 5 min in a 1.5 mL eppendorf tube to create pellets. Pellets have approximately 1.5 × 10^6^ cells per well and then 500 μL culture media containing mNPs (C = 2 mg/mL of each type of mNPs). All the samples, including the control (cells without mNPs), were thermostated at rt and underwent MHT for 10 min until the temperature reached the desired limits (43–44 °C). After completing the MHT treatment, the supernatant was removed to eliminate any mNPs that had not internalized or surface-attached to the cancer cells. Cells were then resuspended and re-seeded in culture plates to perform an ΜΤΤ assay and Prussian blue staining [20].

### 2.10. Statistics

Results are expressed as means ± SD. Multiple comparisons were performed using the Student’s *t* test or one-way or two-way ANOVA using GraphPad Prism 6. Statistical significance wasaccepted at * *p* < 0.05 and ** *p* < 0.001.

## 3. Results and Discussion

### 3.1. Iron Oxide Magnetic Nanoparticles (mNPs): Types and Morphology

Iron oxide magnetic nanoparticles were synthesized, as reported in our previous work, with the co-precipitation method [13]. mNP preparation was based on the co-precipitation methodology due to itslow cost and versatility with regard to obtaining crystalline nanoparticles [21]. In the previous work, three types of mNPs were obtained using this method with ferric and ferrous chloride precursors, having an average size of 5–11 nm. The fabricated mNPsare (i) mNPs (bare mNPs), (ii) citrate-coatedmNPs (mNPs@citrate), and (iii) the one with sodium borohydride (NaBH_4_)/trisodium citrate (mNPs@NaBH_4_). The different reducing and modifying agents were used to improve the colloidal behavior of mNPs, as well as to control their size. mNPs have been characterized as structural, proving their successful coating with the inorganic agents and morphology. The small size that these types of mNPs have is an advantage owing to their easy internalization inside the cells [22].

### 3.2. Evaluation of A375-M6 mNPs Uptake by Electron Microscopy

It has been reported that the size, shape, and charge of magnetic nanoparticles (mNPs) play important roles in their cellular uptake [23,24]. Positively charged mNPs have been shown to exhibit a higher degree of internalization compared to neutral or negatively charged mNPs. This can be attributed to the electrostatic forces that occurred between the mNPs and the cell membrane. This interaction can facilitate mNP attachment and subsequent internalization into the cells [25].

Some studies have reported that positively charged nanoparticles may induce higher cytotoxicity due to their potential to disrupt cell membranes or interact with cellular components. On the other hand, negatively charged nanoparticles are generally considered to have lower toxicity levels, making them more favorable for biomedical applications [14]. It is worth mentioning that the cell membrane can exhibit localized areas of charge due to the presence of charged molecules such asions and specific proteins that are integrated into the membrane structure. For instance, within the cell membrane, there are specialized channels and transporters responsible for controlling the movement of ions such assodium, potassium, and calcium that are positively charged in and out of the cell. These channels and transporters contain regions with electrical charges that aid in the facilitation of ion transport [26]. In our case, negatively charged mNPs seem to exploit this phenomenon to promote internalization through electrostatic interactions.

**Figure 2:**
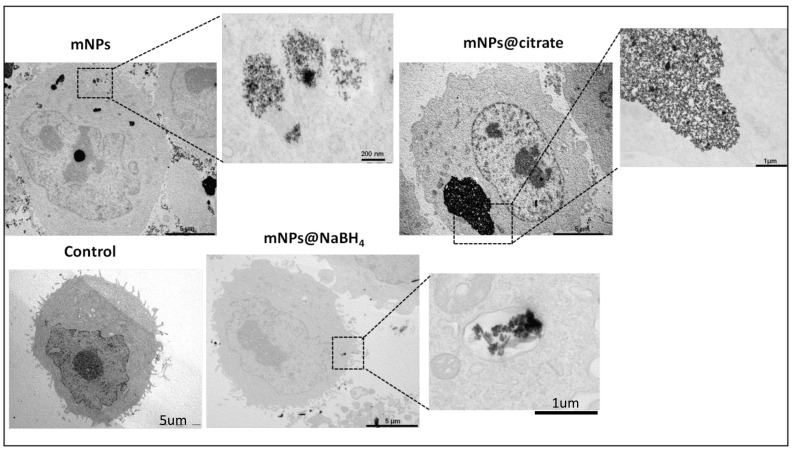
Transmission electron micrographs of A375-M6 treated with the three different types of mNPs for 24 h. In the bottom panel higher magnification images.

As reported in Figure 2, the presence of mNP clusters within the cell cytoplasm after a 24 h treatment suggests that cellular uptake of the nanoparticles has occurred. It appears that all three types of nanoparticles, including citrate-coated, bare, and NaHB_4_-coated nanoparticles, were able to enter the cells and form clusters. The finding that a massive amount of mNPs@citrate and mNPs were accumulated compared to mNPs@NaBH_4_ could indicate differences in their cellular uptake mechanisms or efficiency. For instance, mNPs@citrate are known to have a negative surface charge, which is unfavorable for interactions between the nanoparticles and negatively charged cell membranes. Despite this repulsion, Forest and Pourchez et al. [26] reported the internalization of negatively charged nanoparticles due to cationic sites of cellular membrane allowing the development of electrostatic interactions leading to subsequent internalization. Without any surface coating, mNPs could potentially have more direct contact with the cell membrane, promoting their uptake. As for mNPs@NaBH_4_, the surface chemistry and their size possibly affect their internalization ability.

### 3.3. mNPs Sensitivity by Trypan Blue Assay

**Figure 3:**
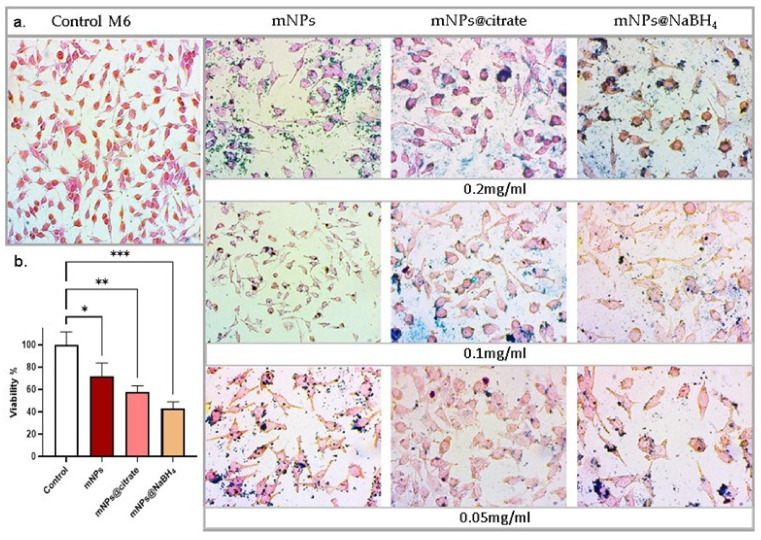
(**a**) Optical microscopy images of cells stained with Prussian blue to detect the presence of iron ions in the cell, (**b**) Trypan blue assay on the melanoma cell line A375-M6. Error bars represent the mean ± standard deviation (SD) from three experiments (Red: mNPs, Pink: mNPs@citrate, Orange: mNPs@NaBH_4_). The asterisk denotes a significant difference compared to untreated cells (* *p* < 0.0001, ** 0.001 < *p* < 0.01, *** 0.0001 < *p* < 0.001). (Scale bar: 10 μm).

Since in our previous paper [13] the mNPs tested at all different concentrations did not cause obvious toxicity of NCTC 2544, we evaluated cell viability of cancer cells (A375-M6) with the three types of mNPs at the highest concentration (0.2 mg/mL) for 24 h. As shown in Figure 3b, the treatment induced a significant reduction in the cell viability of A375-M6 and morphology changes such as cell shrinkage (Figure 3a), which is a characteristic feature of cell death. The optical images stained with Prussian blue, which detects the iron ions, provide visual evidence of these changes (Figure 3b). According to our results, we can indicate that the mNPs@citrate experienced a higher reduction in cell viability and higher levels of cell shrinkage, confirmed by the Prussian blue protocol. Based on that, Prussian-stained nanoparticles in the optical images provide strong evidence in support of the internalization vs. toxicity profile in the A375-M6 cells. The figures for treated cells represent fully stained nanoparticles in the internal area and on the cell surface. These results are in good agreement with the results in Section 3.2.

### 3.4. Apoptotic Evaluation by Confocal Microscopy and ICP-AES Analysis

**Figure 4:**
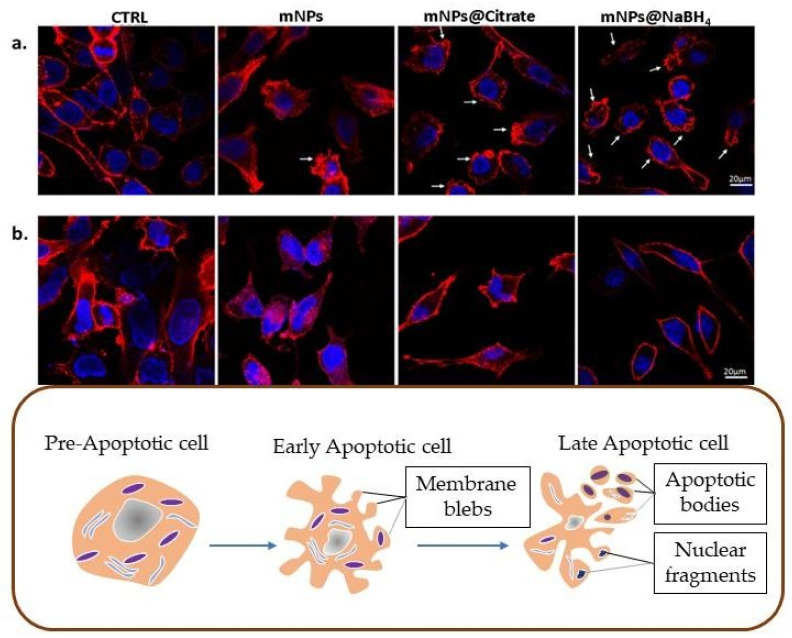
Fluorescence microscopy of (**a**) A375-M6 cells and (**b**) of NCTC 2544, treated with mNPs and labeled with phalloidin (RED), which stains cytoskeletal F-Actin.

To investigate whether the mNP-induced growth inhibition and the cytotoxic effects on melanoma cells were due to programmed cell death, confocal microscopy was performed by staining F-Actin with phalloidin to evaluate cytoskeletal damage. It is well known that cell structure integrity can be evaluated by actin filaments with phalloidin. Membrane blebbing, or rather bubble-like protrusions on the cell surface, is a morphological alteration of cell damage, featuring early apoptosis. Confocal microscopy on A375-M6 revealed (Figure 4a) a blebbing onset of the plasma membrane induced by bare mNPs and intensive blebbing activity in the presence of mNPs@citrate and mNPs@NaBH_4_. No notable variations in the cytoskeletal structure were detected when NCTC 2544 cells were exposed to the three distinct mNPs, meaning that mNPs leave this type of healthy cells unaffected (Figure 4b).

**Figure 5:**
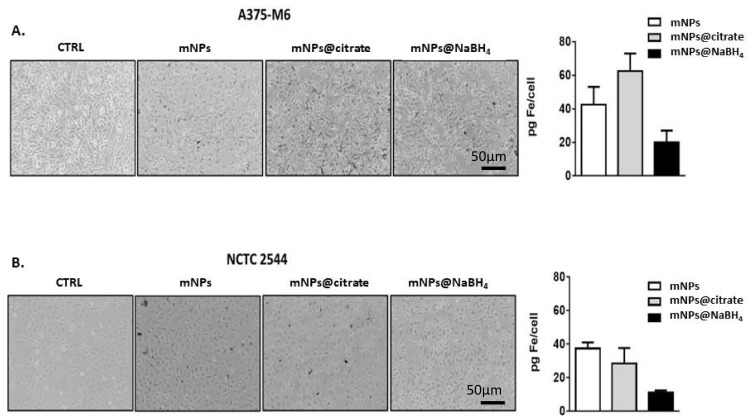
Optical microscopy and ICP analysis of (**A**) A375-M6 cells and (**B**) of NCTC 2544, treated with mNPs, mNPs@citrate, and mNPs@NaBH_4_.

To support these unquestionable results, the amount of internalized iron was evaluated by using optical microscopy (Figure 5A,B left panel) and inductively coupled plasma-atomic emission spectroscopy (ICP-AES) (Figure 5A,B right panel). The results of the ICP-AES analysis reported in the histograms in Figure 5A,B, as pg of iron per cell, reveal that the two cell lines internalized comparable amounts of mNPs and mNPs@NaBH_4_, while higher levels of mNPs@citrate were observed in A375-M6 cells. Optical images in both panels show internalized mNPs as black areas inside the cells, results that are confirmed and quantified by ICP analysis.

### 3.5. Apoptotic Evaluation by Western Blot Analysis

**Figure 6:**
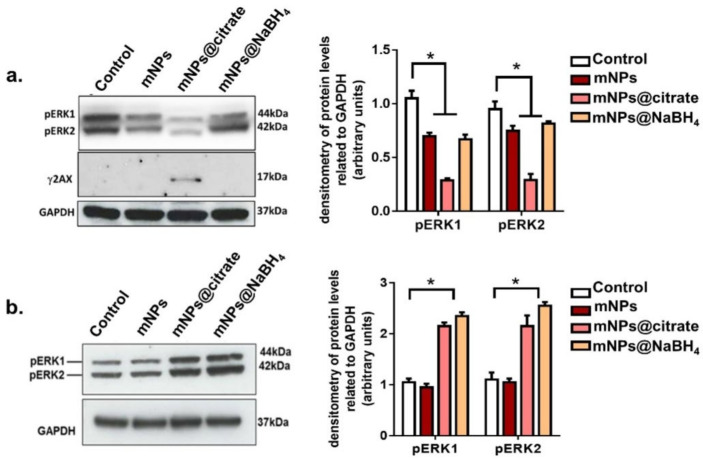
Western blot analysis of (**a**) γH2AX and ERK1/2 phosphorylation levels in A375-M6 and (**b**) NCTC 2544 with relative densitometric quantification normalized GAPDH used as a loading control. Error bars represent the mean ± standard deviation (SD) from three experiments, with statistical significance denoted by * *p* < 0.05 (One star means *p* < 0.05).

Cell survival and death can be evaluated by studying many protein kinases. A375-M6 cells harbor the V600E BRAF mutation which is associated with the mitogen-activated protein kinase (MAPK) signaling pathway resulting in ERK1/2 phosphorylation [17]. This process activates the nucleus and phosphorylates a plethora of substrates that stimulate cell proliferation. The activation of ERK1 inhibits mitochondrial permeability, leading to the inhibition of mitochondrial apoptotic pathways [27]. To further investigate the mechanism involved in the induction of apoptosis in A375-M6 cells, ERK and H2AX phosphorylation levels were studied upon treatment with all mNPs. H2AX phosphorylation resulted inγH2AX, which is a specific marker for DNA double-stranded breaks involved in damaged DNA repair and responsible for the degradation of DNA leading to cell death [28,29]. Western blot images and analyses showed a significant decline in ERK1 phosphorylation induced by all the mNPs (Figure 6a). Nevertheless, a greater reduction inERK1/2 phosphorylation was observed upon mNPs@citrate treatment, along with noticeable levels of H2AX phosphorylation. No detectable γH2AX protein levels were perceived after mNPs and mNPs@NaBH_4_, as well as after all mNPs treatment on NCTC 2544. It should also be noted that ERK1/2 phosphorylation in NCTC 2544 cells was unaffected by mNP treatment, whereas mNPs@citrate and mNPs@NaBH_4_ induced a strong increase in phosphorylation (Figure 6b and Figure S1). Notably, western blot analysis, along with the cell viability and confocal data, confirms the major toxic effects of mNPs on melanoma cells A375-M6, while healthy cells were found to remainremarkably unscathed even though they incorporated a fair amount of mNPs.

### 3.6. FACS Analysis of Sub-Cycling Cell Fraction Using Propidium Iodide Staining

**Figure 7:**
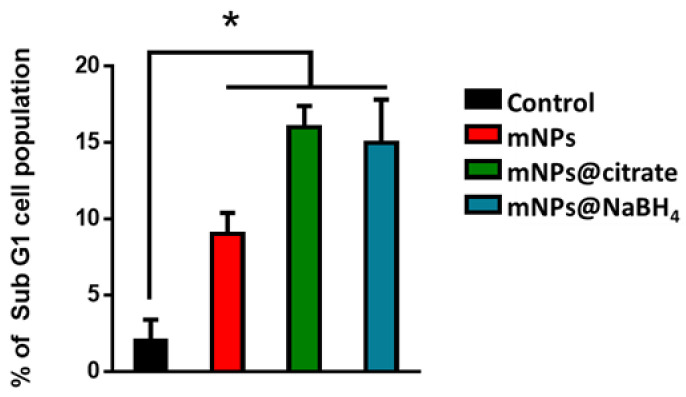
Presentation ofA375-M6 cell percentages in the sub-G1 phase by PI assay. Significance was assessed by a one-way ANOVA test followed by the Newman–Keuls posttest. Error bars represent the mean ± standard deviation (SD), while asterisks (* one star means *p* < 0.05) highlight a significant distinction between the untreated M6 cells (CTRL).

To evaluate the apoptosis in different cell models through flow cytometry, propidium iodide (PI) was used. The PI assay can identify apoptotic cells that are characterized by DNA fragmentation and, consequently, loss of nuclear DNA content. The apoptotic effect of mNPs on A375-M6 cells was confirmed by FACS analysis (Figure 7) with an augmentation of the cells in the sub-G1 phase indicative of cell death. In particular, we observed a higher percentage of apoptotic cells after the treatment with mNPs@citrate and mNPs@NaBH_4_. No difference was observed between untreated and treated NCTC 2544.

### 3.7. In Vitro Magnetic Hyperthermia Treatment and Its Effects on Cell Death with Western Blot

**Figure 8:**
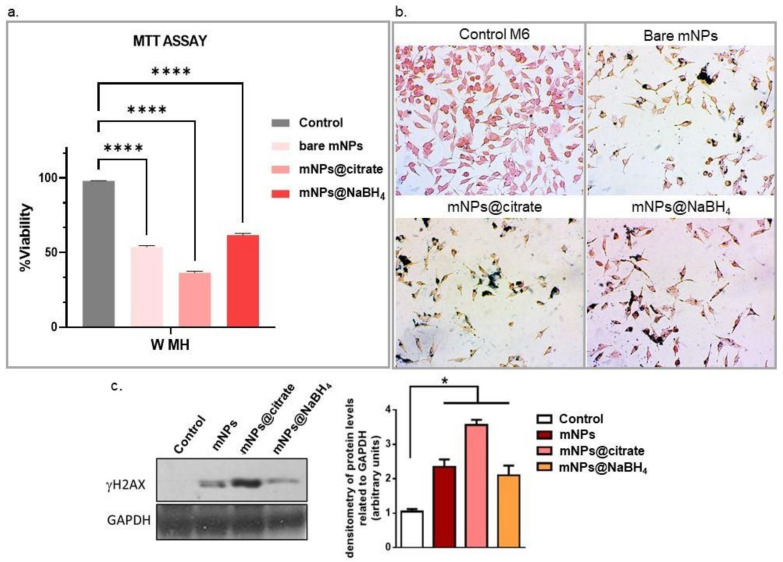
In vitro MH (**a**) MTT viability assay. Statistical analysis by two-way ANOVA test with Dunnett’s multiple comparisons tests (One star means * 0.01 < *p* < 0.05, and the sign with four stars means **** 0.0001 < *p*< 0.001) and (**b**) cell images of Prussian-blue-stained cells. (**c**) Western blot analysis of γH2AX phosphorylation levels in A375-M6 after AMF stimulation. GAPDH is used as a loading control. Scale bar: 10 μm. Error bars indicate mean ± SD; n = 3 experiments; (One star means * *p* < 0.05).

The effectiveness of magnetic nanoparticle-based hyperthermia was then evaluated by assessing cell viability with MTT and by conducting molecular analysis of DNA damage by western blot after exposing the untreated and mNPs-treated cells to AMF, as reported in Section 2. Figure 8a clearly shows a pronounced effect of the magnetic field on cell viability, leading to a substantial reduction in the cell population. Specifically, more than 50% of the cells treated with all the tested mNPs died following the magnetic hyperthermia treatment. In addition to the quantitative assessment of cell viability, cell loss was also confirmed by visual examination of cells after blue Prussian staining (Figure 8b). The optical microscopy images provide visual evidence supporting the impact of the magnetic field on mNP-treated cells. This complements the quantitative cell viability data, providing a comprehensive understanding of the cytotoxic effects induced by the magnetic hyperthermia treatment on cells that incorporate mNPs (cells Prussian blue positive).

The efficacy of the magneto-mechanical potential of mNPs wares was also confirmed by western blot analysis of H2AX phosphorylation levels (Figure 8c). Results in Figure 8b show that exposure to the magnetic field significantly upregulates phosphorylated H2AX levels after mNPs@citrate treatment, while inducing the H2AX phosphorylation in the presence of mNPs and mNPs@NaBH_4_.

## 4. Conclusions

This study provides a comprehensive understanding of the effects of magnetic nanoparticles (mNPs) on cell viability, morphological changes, and molecular alterations, as well as ofthe potential for magnetic-hyperthermia-induced cytotoxicity. These findings offer valuable insights into the mechanisms underlying the impact of mNPs on both cancer cells (A375-M6) and healthy cells (NCTC 2544), as well as into their potential applications in cancer therapy. Intriguingly, prior research [13] indicated that mNPs did not cause obvious toxicity to NCTC 2544 cells across various concentrations.

Building upon this foundational knowledge, the current study focused on evaluating the cell viability of A375-M6 cancer cells when exposed to three types of mNPs at their highest concentration (0.2 mg/mL) for 24 h. The results demonstrated a significant reduction in the cell viability of A375-M6 cells, accompanied by noticeable morphological changes, including cell shrinkage, a hallmark of cell death. The use of Prussian blue staining provided visual evidence of these alterations, further supporting the observation that mNPs@citrate exhibited the most pronounced reduction in cell viability and cell shrinkage compared to the other mNPs tested. This can be ascribed to the favorable colloidal properties exhibited by the particular sample. The confocal microscopy, employed to assess cytoskeletal damage, underscores the selectivity of mNPs for cancer cells that exhibit plasma membrane blebbing, a characteristic feature of early apoptosis, while no significant differences in the cytoskeletal structure were observed in NCTC 2544 cells exposed to any of the three mNPs. Flow cytometry analysis confirmed the apoptotic effect of mNPs on A375-M6 cells, with higher percentages of apoptotic cells observed after treatment with mNPs@citrate and mNPs@NaBH_4_. Importantly, no significant differences were noted between untreated and treated NCTC 2544 cells, underscoring the safety of mNPs for healthy cells. Furthermore, the efficacy of magnetic hyperthermia was assessed, revealing a substantial reduction in cell viability following exposure to the magnetic field, particularly in cells treated with mNPs. Visual examination, using Prussian blue staining, supported the quantitative cell viability data, highlighting the cytotoxic effects of magnetic hyperthermia on mNP-incorporated cells. Western blot analysis of H2AX phosphorylation levels further confirmed the magneto-mechanical potential of mNPs.

In summary, these comprehensive results provide strong evidence supporting the potential therapeutic use of mNPs, particularly mNPs@citrate, for inducing cytotoxicity in cancer cells while sparing healthy cells. The findings highlight the selectivity, internalization, and molecular mechanisms underlying the apoptotic effects of mNPs, paving the way for further exploration of their application in cancer therapy, including magnetic-hyperthermia-based treatments.

This study offers a multifaceted perspective on the interactions between magnetic nanoparticles (mNPs) and different cell types, shedding light on their potential applications in cancer therapy, their selectivity, internalization mechanisms, and their role in programmed cell death. This discussion will provide an overview of the key findings and their implications.

## Data Availability

Data are contained within the article and Supplementary Materials.

## Supplementary Materials

The following supporting information can be downloaded at: https://www.mdpi.com/article/10.3390/pharmaceutics16040527/s1, Figure S1: Gels and blots from Western blot analysis.
