# Supplementary material for: Iron Oxide Nanoparticles: Selectively Targeting Melanoma Cells In Vitro by Inducing DNA Damage via H2AX Phosphorylation and Hindering Proliferation through ERK Dephosphorylation
Source: Pharmaceutics. 2024 Apr 11;16(4):527. doi: 10.3390/pharmaceutics16040527 (PMC11054682; doi:10.3390/pharmaceutics16040527)
Supplement: Supplementary file 1 [file pharmaceutics-16-00527-s001.zip › pharmaceutics-2612606-supplementary.pdf]

Supporting Information:

Title: Cells In Vitro by inducing DNA Damage via H2AX Phosphorylation and hindering proliferation through ERK dephosphorylation

Danai Prokopiou E.[1,2], Anastasia Chillàc[3], Francesca Margheri[3], Gabriella Fibbi[3], Anna Laurenzana[3*], Eleni Efthimiadou K.[1,2*]

[1]Inorganic Chemistry Laboratory, Chemistry Department, National and Kapodistrian University of Athens, Panepistimiopolis, Zografou 157 71, Greece; dprokop@chem.uoa.gr, efthim@chem.uoa.gr

[2]Institute of Nanoscience and Nanotechnology, NCSR "Demokritos", 153 41 Aghia Paraskevi Attikis, Greece; dprokopiou@inn.demokritos.gr

[3]Department of Experimental and Clinical Biomedical Sciences "Mario Serio", University of Florence, Viale G.B. Morgagni, 50, Florence 50134, Italy; anastasia.chilla@unifi.it, francesca.margheri@unifi.it, gabriella.fibbi@unifi.it, anna.laurenzana@unifi.it

*Correspondence: A.L. anna.laurenzana@unifi.it; Tel: +39 055 2751300, E.E. efthim@chem.uoa.gr, Tel: +302107274858

Since our collaborators in University of Florence use the tested antibodies routinely, they know the properties of the Ab very well, they cut the single membranes so that each cut membrane only contains the molecular weight protein of interest and then they did single Ab WBs on each. For the GAPDH staining, (very molecular weight very close to ERK1/2 the membrane was stripped and re-probed). For γH2AX on A375 lysates a longer exposure was performed.

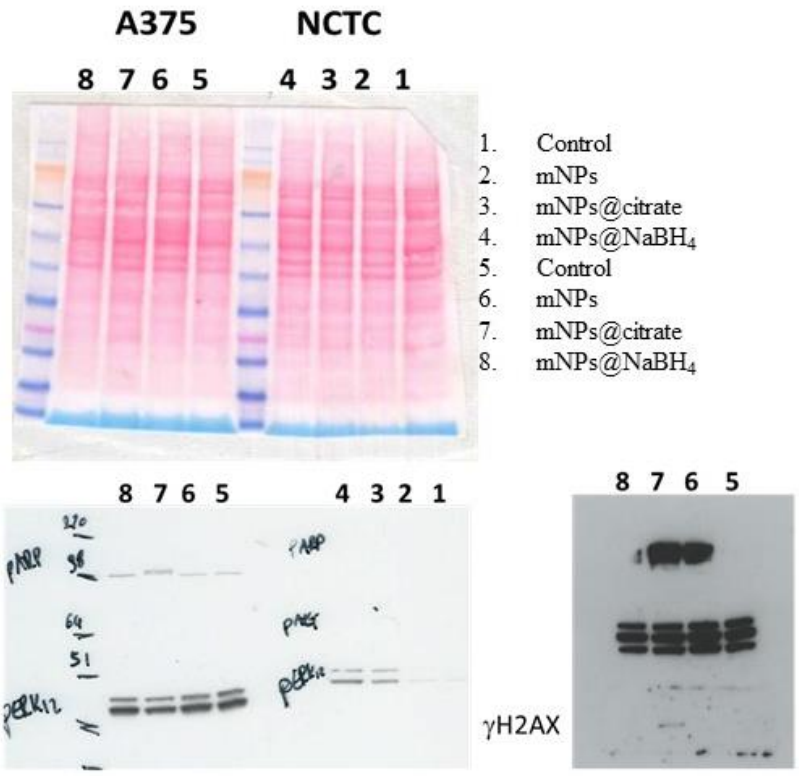

Figure S1. Gels and blots from Western blot analysis.
